# Notes and Notices

**Published:** 1892-05

**Authors:** 


### NOTES AND NOTICES.

The Homœopathic Medical College of Missouri held its 33d annual commencement exercises at Pick Wick Theatre, St. Louis, on the evening of March the 17th, and graduated seven M. D's. Owing to the rigorous adhesion to the three years' course of study, there were not more, but in consideration of the fact that the older colleges of the dominant school only graduated from fifteen to twenty, the management feel satisfied with the work of the term past.

The exercises were interspersed with the vocal and instrumental selections of the best musicians of the city; the address on behalf of the faculty was delivered by Rev. J. J. O'Brien, subject, " The elements of success," and the degree of Doctor of Medicine was conferred upon Ferdinand Brase, Helene A. Goerke, Clara Louise Toby, Thos. J. Jones, Emma C. F. Wentzel, E. Wilson

Taylor, and Paul N. Zilliken, by the President of the Board of Trustees, Dr. W. A. Edmonds; Professor I. D. Foulon awarded the prizes and flowers in his usual happy style, and following the benediction the large audience and the graduates filed out to assume each his or her duty on the next day.

The Cleveland Homœopathic Medical College (the new college) held its second annual commencement on Wednesday evening, March 23d, 1892.

In the absence of Judge W. W. Boynton, the President of the college, Vice-President White conferred the degree of Doctor of Medicine upon the following graduates: Herman C. Galster, Homer Bryan, John Melvin Wallace, Harry Louis Sexton, Henry Franck, B. A., Helen Babcock, Hannah Burroughs Mulford, Arthur Besemer, Andrew D. Smith, Howard Burhanse Besemer, Ph. B., M. D., Philip Henry Sigrist, B. S., John L. Winslow, M. D., Ruth Beckwith Kirch, M. E., Ben Wilgus Genung, Henry Lewis Stem, Harriet Warner Carman, A. D., J. Elmer Moore, Frank. W. Somers, George H. Cole, Monroe Manges, A. M., George H. Bradt, Albert E. McClure, H. Josephine Wright, Cornelius Crosby Albert.

A New Materia Medica.—Drs. Rufus L. Thurston and Samuel A. Kimball, of Boston, are about publishing a new *Materia Medica* which they hope will be an improvement on anything heretofore issued. They have found that no one book of materia medica has all the symptoms that belong to the different remedies. They, therefore, propose to collect together in one volume the symptoms of about two hundred remedies and make the volume of convenient size for use at the bedside. Every homœopathic physician should subscribe to this work and ensure its early completion. Send for explanatory circular to Dr. S. A. Kimball, 124 Commonwealth Ave., Boston, Mass.

The Homœopathic Medical Society of Ohio will hold its annual meeting May 10th and 11th, at Cincinnati. The President, Dr. Charles D. Crank, is out in an urgent appeal for a full attendance of the members.

The World's Columbian Exposition.—Send fifty cents to Bond & Co., 576 Rookery, Chicago, and you will receive, post-paid, a four-hundred page advance *Guide to the Exposition*, with elegant engravings of the grounds and buildings, portraits of its leading spirits, and a map of the city of Chicago; all of the rules governing the Exposition and exhibitors, and all information which can be given out in advance of its opening. Also, other engravings and printed information will be sent you as published. It will be a very valuable book and every person should secure a copy.

Dr. J. M. Selfridge, of Oakland, California, in March last, passed the fortieth anniversary of his graduation as a homœopathic physician. We cordially hope the worthy doctor will see many more such anniversaries. Dr. Selfridge is an ardent homœopathist.

The Cleveland Homœopathic Hospital College calls attention to the following facts:

1. The Post-Graduate Course, March 28th, continuing two weeks, will offer unusual advantages for clinics. 2. The college will give free surgical treatment, to cases you consider deserving, during the course. 3. The Gynæcological and General Surgical Clinic on Thursday, 2 to 4 P. M., and General Orthopedic Surgical Clinic on Wednesdays, from 10 A. M. to 12 M., at College and daily operations at hospital from 12 to 2 P. M. 4. Have you not a case that you can send, or better bring with you for the two weeks Post-Graduate course? 5. The clinics this year have been unprecedented, both as regards number and value. The classes watching the conservatism of homœopathic treatment of surgical cases, as well as seeing the more daring operations, have had an especial advantage, in that each one has had opportunity to practice the *technique* of *dressings,* so valuable to a beginner. 6. The excavation for the new college building is under way. Architect and contractors promise occupancy September 1st, 1892. 7. If you bring or send a case, please address us at 176 Euclid Ave. H. F. BIGGAR, *Prof. of Gynæcology, Operative and Clinical Surgery.*

NEW YORK PASTEUR INSTITUTE, for the preventive treatment of hydrophobia and for the study of contagious diseases, 178 West 10th Street, has rendered its second annual report, from which are derived the following conclusions:

By the examination of the figures given, one may see that the results obtained at the New York Pasteur Institute are about the same as those reported by the kindred institutions. It is unnecessary to comment upon them, and they will be well appreciated by any unprejudiced mind.

Let us remember that last year forty-two deaths caused by hydrophobia have been formally reported of persons bitten by rabid animals, and who were not submitted to the inoculations. This makes about eighty deaths for the period included in our statistics, during which three persons died despite the treatment. And without taking an exception for the patient (Earl) who came only four days after his terrible fight with the dog, if we consider as they do abroad that this treatment has produced its full effects only fifteen days after it had been completed, we see that the percentage of those who died after the fifteen days following the inoculation has been only two out of two hundred and ninety-eight or sixty-six one-hundreths per cent.

Among the two hundred and ninety-eight persons treated, one hundred and seventy-seven have been attacked by animals undoubtedly rabid; if we consider, again, that statistics indicate twenty-five per cent. as a low percentage of deaths after bites inflicted by hydrophobic dogs; and, then counting only one hundred and seventy-seven instead of two hundred and ninety-eight persons bitten, we ought to have had not two or three cases of death, but at least forty-four. Moreover, one hundred and twenty-three persons among the one hundred and seventy-seven bitten by hydrophobic animals had their wounds inflicted on the face, the head, or the hands. We know that bites of this nature are followed by hydrophobia in a much larger proportion, say at least forty per cent. The number of deaths ought to be, then, no less than *seventy* among the persons so bitten who came from the different parts of the country to submit themselves to the Pasteur treatment.

